# KIF7 attenuates prostate tumor growth through LKB1-mediated AKT inhibition

**DOI:** 10.18632/oncotarget.17421

**Published:** 2017-04-26

**Authors:** Kai Yau Wong, Jing Liu, Kwok Wah Chan

**Affiliations:** ^1^ Department of Pathology, The University of Hong Kong, Hong Kong

**Keywords:** KIF7, tumor suppressor gene, prostate cancer, LKB1

## Abstract

This study investigated kinesin family member 7 (KIF7) expression and function in prostate cancer (PCa). Our results showed that KIF7 was significantly downregulated in PCa, compared with normal, benign prostatic hyperplasia and prostate intraepithelial neoplasia tissues, partially through promoter hypermethylation. We further investigated the effects of KIF7 coiled coil (CC) domain and motor domain (MD) on PCa development *in vitro* and *in vivo*. Our results showed that KIF7-CC but not KIF7-MD significantly attenuated proliferation and colony formation, impeded migration and invasion, induced apoptosis and sensitized PCa cells to paclitaxel. Further analysis revealed that KIF7-CC enhanced LKB1 expression and phosphorylation at Ser^428^, which induced PTEN phosphorylation at Ser^380^/Thr^382/383^ and consequently blocked AKT phosphorylation at Ser^473^. Downregulation of LKB1 significantly attenuated the suppressive effects of KIF7-CC on cell proliferation, colony formation and AKT phosphorylation. Furthermore, our *in vivo* studies showed that KIF7-CC reduced prostate tumorigenesis in cell-derived xenografts. Downregulation of LKB1 abrogated the anti-tumor effects of KIF7-CC in these xenografts. Taken together, these findings provide the first evidence to support the role of KIF7 as a negative regulator that inhibits PCa development partially through LKB1-mediated AKT inhibition.

## INTRODUCTION

Prostate cancer (PCa) is the most common cancer in men and is the second leading cause of cancer death worldwide [1, 2]. In 2015, 220,800 new cases of prostate cancer are found accounting for about one-quarter of new diagnoses in the United State. This means one of seven men is being diagnosed with prostate cancer over their lifetime. About 27,540 men die each year from prostate cancer-related illness [2]. Although androgen deprivation therapy tends to be the first-line treatment, most patients inevitably develop castration-resistant PCa 14-20 months after castration [3]. Chemotherapy with taxane is one of the few therapeutic options for castration-resistant PCa [4]. Unfortunately, resistance to taxane treatments severely limits the potential of these agents to improve the lives of patients [5]. Hence, a better understanding of the molecular mechanisms of PCa progression will develop more effective targets for diagnosis and enhance therapeutic responses to taxane-based therapy.

Obesity facilitates prostate carcinogenesis and progression as well as increases the risk of therapeutic treatment failure [6, 7]. Dysfunction of primary cilia has been shown to be linked to obesity and neoplasms [8, 9]. Absence of the cilium can be an initiating event for neoplastic growth [10], especially in PCa [11]. Kinesin family member 7 (KIF7) on 15q26.1 is a Kinesin-4 family member that has been shown to play critical roles in primary cilia formation and Hedgehog (Hh) signaling in embryonic development [12, 13]. Loss of 15q26.1 to 15q26.3 has been found to be related to colorectal carcinoma [14]. However, the expression and role of KIF7 in human solid tumors still remains unknown.

In the present study, down-regulation of KIF7 was associated with promoter hypermethylation in PCa. KIF7-mediated attenuation of PCa development was abrogated by LKB1 inhibition. Our results reveal that KIF7 is a novel tumor suppressor in PCa that acts by suppressing proliferation, migration, invasion and tumorigenicity through LKB1/PTEN/AKT signaling pathway.

## RESULTS

### KIF7 as a novel tumor suppressor gene in human PCa

**Figure 1:**
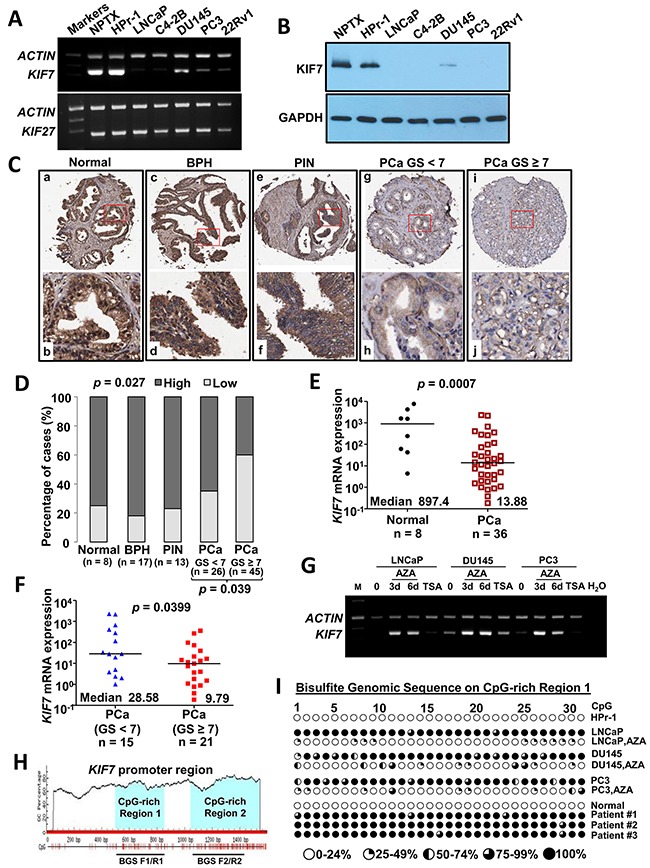
KIF7 was downregulated in prostate carcinogenesis through promoter hypermethylation **(A)** multiplex RT-PCR analysis of *KIF7* and *KIF27* from normal prostate epithelial cells (NPTX and HPr-1) and PCa cells (LNCaP, C4-2B, DU145, PC3 and 22Rv1). **(B)** KIF7 in different PCa cells compared with normal epithelial cells by western blot. **(C)** IHC staining of KIF7 in the human prostate TMA. (a&b) Normal prostate; (c&d) BPH; (e&f) PIN; (g&h) low-grade PCa and (i&j) high-grade PCa. **(D)** bar-char summarizing the IHC of KIF7 in non-tumor *vs*. PCa specimens. **(E)** *KIF7* expression in normal prostate and PCa by qPCR. **(F)** *KIF7* expression in low-grade and high-grade PCa by qPCR. **(G)** *KIF7* expression in LNCaP, DU145 and PC3 cells treated with 5-AZA-dC or TSA by multiplex RT-PCR. **(H)** two CpG-rich regions from *KIF7* promoter were predicted by Methprimer CpG Island Prediction program (http://www.urogene.org/cgi-bin/methprimer/methprimer.cgi). **(I)** BGS of the *KIF7* CpG-rich region 1 in HPr-1, LNCaP, DU145 and PC3 cells treated without or with 5-AZA-dC (3 d) as well as one normal prostate and three PCa specimens.

KIF7 and KIF27 (9q22.1) are paralogues that share 44% sequence identity overall and have even higher identity in the motor domain (MD) (61%). To investigate the expression of KIF7 and KIF27 in PCa, mRNA expression of these two genes was detected by multiplex reverse transcription PCR (RT-PCR) in two immortalized normal prostate epithelial cell lines HPr-1 and NPTX and five PCa cell lines including LNCaP, C4-2B, DU145, PC3 and 22Rv1. The mRNA expression in each sample was normalized to *β-ACTIN*. *KIF7* was highly expressed in two immortalized normal prostate epithelial cell lines but frequently downregulated or silenced in PCa cell lines. No difference was found in *KIF27* expression (Figure 1A). Consistent results were found in KIF7 protein levels *in vitro* (Figure 1B). These data showed that KIF7 but not KIF27 might play a suppressive role in PCa.

To investigate the expression of KIF7 and its clinical significance in PCa, immunohistochemistry (IHC) of KIF7 was performed on our human prostate tissue micro-array (TMA). The PCa group was classified into low (GS < 7, *n* = 26) and high (GS ≥ 7, *n* = 45) grades, based on their combined Gleason score (GS) (Supplementary Table 1). KIF7 was highly expressed in the normal prostate (Figure 1C a&b), benign prostatic hyperplasia (BPH) (Figure 1C c&d) and prostate intraepithelial neoplasia (PIN) (Figure 1C e&f). Within the PCa (*n* = 71), moderate to weak signals were detected in the well-differentiated cancerous glandular epithelia, with decreasing expression detected from low-grade (Figure 1C g&h) to high-grade PCa (Figure 1C i&j). Negative to weak staining of KIF7 proteins was detected in 8 out of 38 (21%) non-tumors and 36 out of 71 (51%) tumors, of which 9 were found in low-grade and 27 in high-grade PCa. Moderate to strong staining was detected in 30 out of 38 (79%) non-tumors and 35 out of 71 (49%) tumors, of which 17 was found in low-grade and 18 in high-grade tumors. A χ^2^ test revealed a significant correlation in KIF7 expression between non-tumors and tumors (*p* = 0.027) as well as between the low-grade and high-grade PCa (*p* = 0.039) (Figure 1D). KIF7 expression was not correlated with prostate specific antigen levels (*p* = 0.763), metastasis (*p* = 0.919) or the mean age (76 years) of the patients (*p* = 0.288) (data not shown). To confirm *KIF7* expression in our clinical tissues, RNA was isolated from formalin-fixed paraffin-embedded (FFPE) PCa and normal tissues. Except for the histological diagnoses made by a pathologist (KW Chan), we have performed the immunohistochemistry of p63 on the blocks after microdissection. p63 is a basal cell marker which was lost in prostate adenocarcinoma [15, 16]. We found that p63 was highly expressed in normal prostate epithelium, while rarely expressed in prostate adenocarcinoma cells (Supplementary Figure 1), which further confirmed that RNA from tumors were not contaminated by normal tissues. *KIF7* was significantly downregulated in PCa (*n* = 36) by 64.7-fold compared to normal controls (*n* = 8, *p* = 0.0007) (Figure 1E). Furthermore, *KIF7* was deceased in high-grade PCa (*n* = 21) by 2.9-fold compared to low-grade PCa (*n* = 15, *p* = 0.0399) (Figure 1F). We also investigated the *KIF7*-expression levels and recurrence in the Gulzar Z *et al*. database (GSE40272) [17]. Tumors with lower *KIF7* expression had a worse recurrence-free survival after radical prostatectomy (Supplementary Figure 2A, *p* = 0.069, log rank test). Additionally, consistent data were found in the Oncomine database, and *KIF7* is significantly downregulated in 3 different PCa studies. Further, down-regulation of *KIF7* is not only restricted to PCa but also present in other cancer types such as breast, renal, melanoma, lung, colorectal, oral, bladder urothelial and gastric cancers as well as vulvar intraepithelial neoplasia (Supplementary Figure 3). Taken together, these results indicate that KIF7 is downregulated and might play a suppressive role in PCa.

### The KIF7 promoter region was frequently hypermethylated in PCa

Epigenetic regulation, including DNA hypermethylation and histone deacetylation, might be involved in the down-regulation of tumor suppressor genes [18]. To determine whether *KIF7* downregulation was associated with epigenetic regulation in PCa, LNCaP, DU145 and PC3 cells were treated with a DNA methyltransferase inhibitor (5-AZA-2’-deoxycytidine, 5-AZA-dC) or a histone deacetylation inhibitor (Trichostatin A, TSA) to investigate the effect of DNA methylation or histone deacetylation on *KIF7* expression. As shown in Figure 1G, *KIF7* expression was restored after demethylation treatments with 5-AZA-dC. Treatment with TSA did not alter *KIF7* expression. These studies support that DNA methylation but not histone deacetylation is involved in *KIF7* inactivation in PCa.

To investigate the role of aberrant promoter hypermethylation in *KIF7* silencing, Bisulfite genomic sequencing (BGS) was conducted to investigate methylation of the *KIF7* promoter region. Two CpG-rich regions of the *KIF7* promoter were predicted by the Methprimer CpG Island Prediction program (Figure 1H). As shown in Figure 1I, no methylation at the *KIF7* promoter CpG-rich region 1 was detected in the KIF7-expressing HPr-1 cells. In contrast, hypermethylation was found in the LNCaP, DU145 and PC3 cells. Compared with their untreated cells, methylation was significantly reduced in these three PCa cells after 5-AZA-dC treatment. In addition, we also investigated the methylation status of one normal prostate [19] and three PCa tissues. Consistent with the methylation status *in vitro*, no methylation was found in the normal tissue, and heavy methylation (75% - 99%) was found in those three PCa tissues. Multiplex RT-PCR result confirmed that lower *KIF7* expression in these three hypermethylated PCa tissues compared with that in the hypomethylated normal prostate (Supplementary Figure 2B). No significant difference was found in the *KIF7* promoter CpG-rich region 2 between the normal and tumor cells (Supplementary Figure 2C). Taken together, these observations indicate that promoter hypermethylation in CpG-rich region 1 is implicated in *KIF7* inactivation in human PCa.

### KIF7-CC domain suppressed *in vitro* proliferation, colony formation, migration, invasion, and sensitized cells to paclitaxel treatment

**Figure 2:**
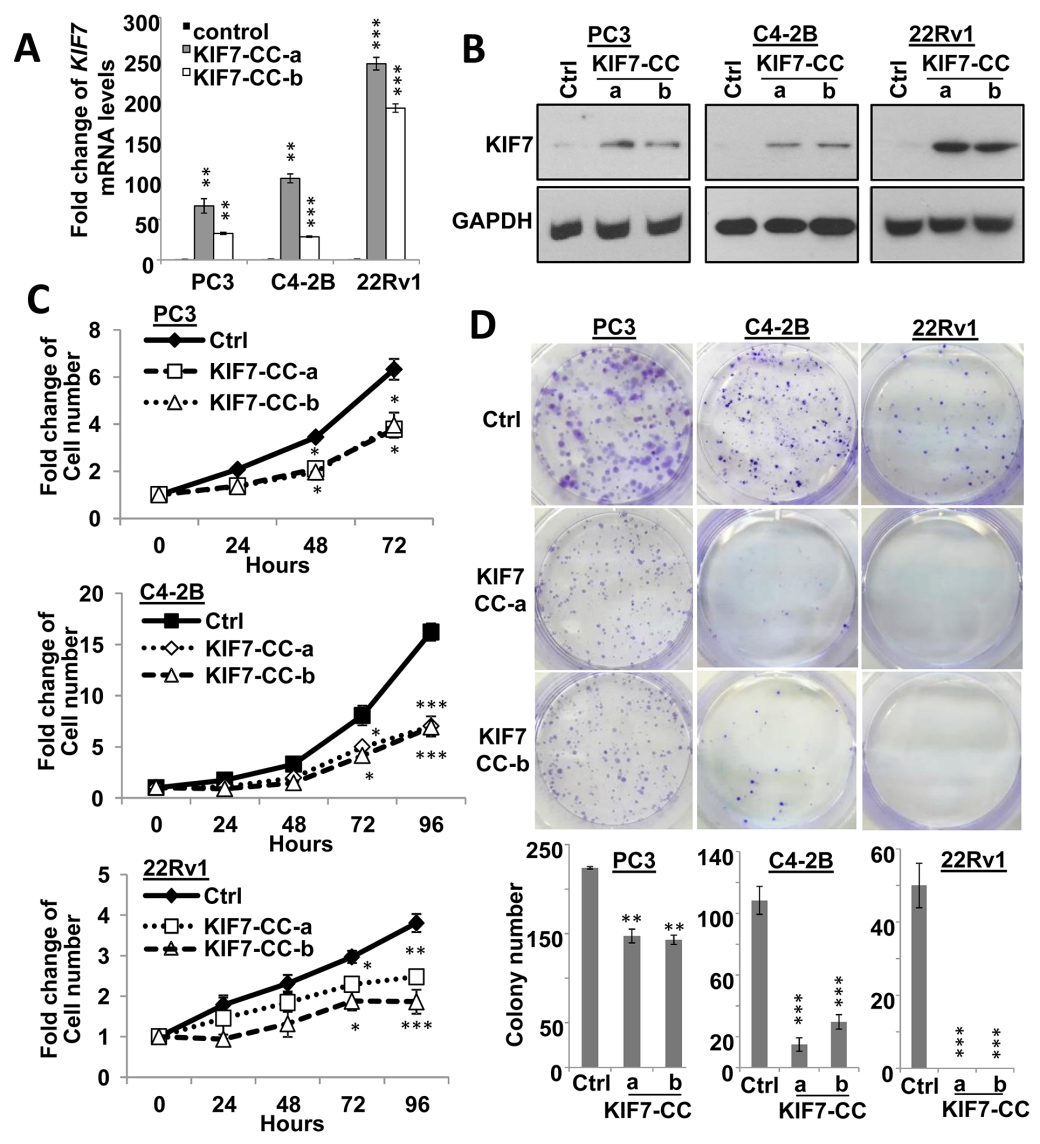
KIF7-CC inhibited proliferation and decreased colony formation in PCa cells KIF7-CC and control vector were stably over-expressed in two different clones from PC3, C4-2B and 22Rv1 PCa cells by qPCR **(A)** and western blot **(B)** analysis. KIF7-CC inhibited cell proliferation **(C)** and decreased colony formation **(D)** in these cells. *, *p* < 0.05; **, *p* < 0.01; ***, *p* < 0.001, mean ± SD was presented.

Epigenetic silencing or downregulation of KIF7 in PCa prompted us to further investigate its functions. KIF7 possesses an N-terminal globular motor domain (MD) that contains nucleotide-binding and microtubule-interacting regions, followed by a stalk domain predicted to form a discontinuous coiled coil (CC) and a globular C-terminal tail domain [20]. Different domains might have different functions, thus we first investigated whether KIF7-CC possessed a tumor-suppressive function in PCa. KIF7-CC was overexpressed in PC3, C4-2B and 22Rv1 cells. Two different stable clones from each cell line were selected and confirmed at both mRNA (Figure 2A) and protein levels (Figure 2B). Overexpression of KIF7-CC inhibited cell growth to 61 ± 1.9%, 43 ± 0.5% and 57 ± 1.4% in PC3, C4-2B and 22Rv1 cells, respectively (Figure 2C). KIF7-CC overexpression significantly decreased colony numbers to 65 ± 1.4% and 21 ± 10% in PC3 and C4-2B cells, respectively. KIF7-CC not only decreased the colony numbers but also inhibited the colony sizes in PC3 cells. Notably, no colony had been formed in 22Rv1 KIF7-CC clones compared with control cells (Figure 2D).

**Figure 3:**
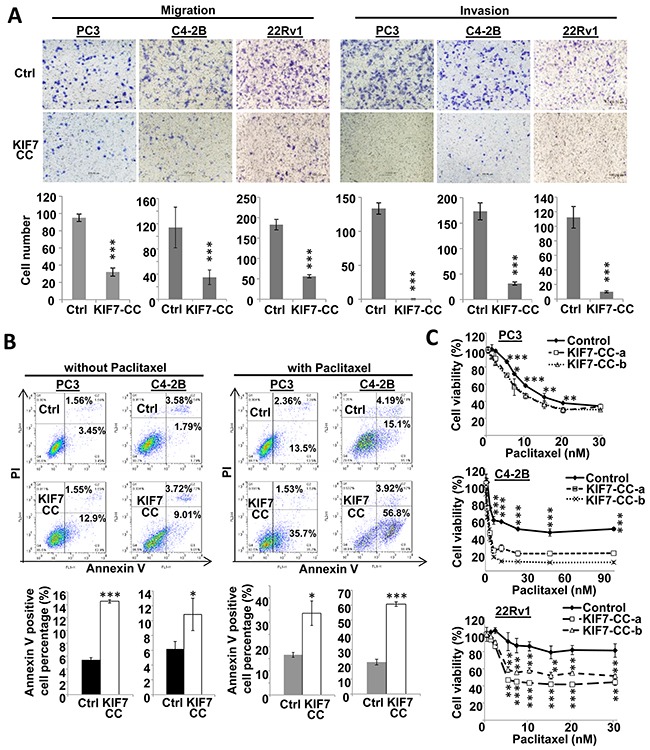
KIF7-CC decreased cell motility and invasiveness, increased cell apoptosis as well as sensitized PCa cells to paclitaxel **(A)** KIF7-CC decreased migration and invasion in PC3, C4-2B and 22Rv1 PCa cells by transwell assay. **(B)** Annexin V-FITC and PI staining showed that KIF7-CC increased apoptosis in PC3 and C4-2B cells compared with vector controls when treated without or with paclitaxel at 5 nM for 3 days. **(C)** drug sensitivity assays showed that KIF7-CC sensitized PC3, C4-2B and 22Rv1 cells to paclitaxel by MTT tests. *, *p* < 0.05; **, *p* < 0.01; ***, *p* < 0.001, mean ± SD was presented.

Stable KIF7-CC overexpression markedly reduced the ability of the cells to migrate and invade. Migration was inhibited to 34%, 31% and 31%, and invasion was decreased to 0.2%, 18% and 9% in PC3, C4-2B and 22Rv1 cells, respectively (Figure 3A). We next tested whether KIF7-CC could induce apoptosis in PCa cells. The results showed that KIF7-CC increased apoptosis from 5.4 ± 0.3% to 14.5 ± 0.3% in PC3 cells (*p* < 0.001) and from 6.2 ± 0.3% to 10.8 ± 2.2% in C4-2B cells (*p* < 0.05) (Figure 3B). Paclitaxel is one of the microtubule-stabilizing drugs that are approved by the U.S. Food and Drug Administration for the treatment of several cancers [21]. We investigated whether KIF7-CC had a synergistic effect with paclitaxel on PCa cell death. The results showed that KIF7-CC significantly increased cell apoptosis from 16.6 ± 1.0% to 33.7 ± 5.0% in PC3 cells (*p* < 0.05) and from 21.6 ± 2.8% to 60.7 ± 1.7% in C4-2B cells (*p* < 0.001) when cells were treated with 5 nM paclitaxel for 3 days (Figure 3B). On the other hand, we treated these cells with different doses of paclitaxel and investigated drug sensitivity. Consistently, KIF7-CC overexpression sensitized the cells to paclitaxel treatment, especially in C4-2B and 22Rv1 cells (Figure 3C). These results suggest an inhibitory role for KIF7 in the regulation of proliferation, migration and invasion as well as increasing the paclitaxel sensitivity of PCa cells.

### KIF7-CC suppressed *in vivo* tumor formation in PCa

**Figure 4:**
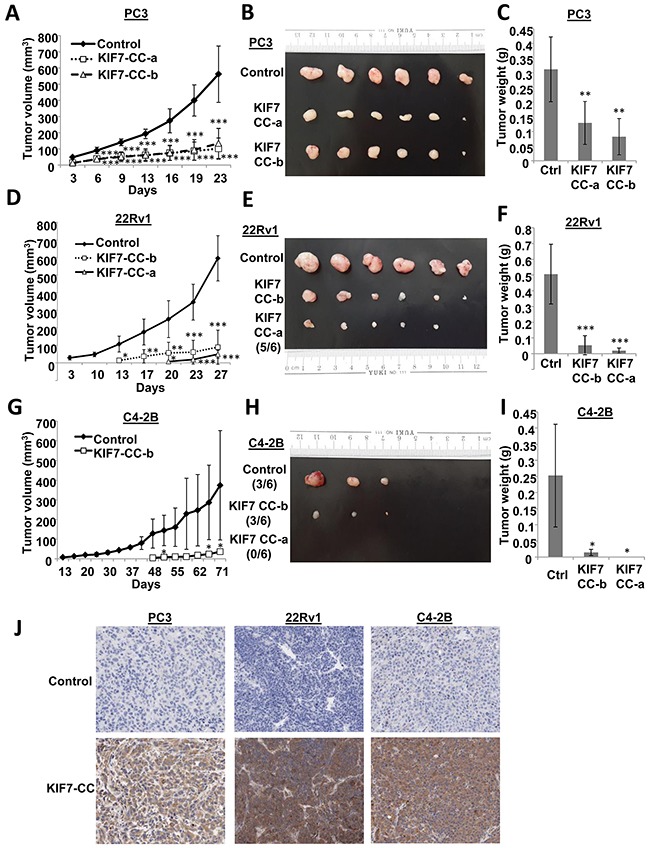
KIF7-CC attenuated PCa cell-derived tumors in nude mice PC3, 22Rv1 and C4-2B cells stably over-expressed KIF7-CC as well as the controls were subcutaneously implanted into the nude mice. **(A, D & G)** tumor growth was monitored at various time points. **(B, E & H)** representative tumor images from PC3, 22Rv1 and C4-2B cell-derived xenografts were shown. **(C, F & I)** bar-char summarized the tumor weights from different groups. **(J)** Immunohistochemistry of KIF7 in PC3, 22Rv1 and C4-2B cell-derived xenografts with or without KIF7 overexpression; *, *p* < 0.05; **, *p* < 0.01; ***, *p* < 0.001, compared with the controls. Data represent the mean ± SD, there are 6 mice in each group.

To support our *in vitro* findings, an *in vivo* nude mouse model was used to evaluate whether KIF7 had any suppressive effects on PCa development. Two different KIF7-CC overexpression clones together with their control cells from the PC3, 22Rv1 and C4-2B cells were injected subcutaneously into the nude mice. As shown in Figure 4, all analyzed KIF7-CC-expressing cells demonstrated lower graft rates (Figure 4A, 4D&4G), smaller volumes (Figure 4B, 4E&4H) and lighter weights (Figure 4C, 4F&4I) compared to the controls. Moreover, KIF7-CC prolonged latency in 22Rv1 and C4-2B xenografts. Overexpression of KIF7 in these xenografts was confirmed by immunohistochemistry (Figure 4J).

### KIF7-MD had no anti-tumor functions in PCa

As KIF7-CC suppressed prostate carcinogenesis both *in vitro* and *in vivo*, we therefore investigated whether KIF7-MD also had anti-tumor functions in PCa. MD-overexpressing (MD) or control cells (GFP) from PC3 and C4-2B were generated (Supplementary Figure 4A). No difference was found on cell proliferation, colony formation or paclitaxel sensitivity in KIF7-MD-overexpressing cells compared with those in the GFP controls from both PC3 and C4-2B cells *in vitro* (Supplementary Figure 4B, 4C, 4D). In addition, we also confirmed that KIF7-MD could not inhibit prostate tumor growth in PC3-derived xenografts *in vivo* (Supplementary Figure 4E, 4F, 4G). These results show that KIF7-MD cannot suppress prostate carcinogenesis.

### Inhibitory effect on prostate carcinogenesis of KIF7-CC might occur through upregulation of LKB1/PTEN

**Figure 5:**
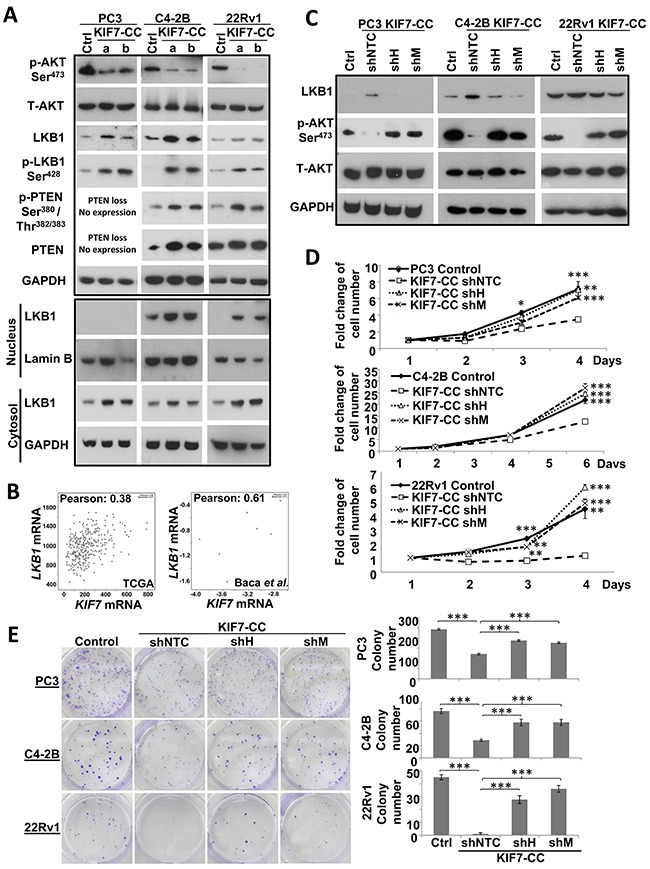
Inhibitory effect of AKT phosphorylation at Ser^473^ by KIF7-CC might partially through upregulation of LKB1/PTEN **(A)** KIF7-CC inhibited AKT phosphorylation at Ser^473^, increased LKB1, LKB1 phosphorylation at Ser^428^ and PTEN phosphorylation at Ser^380^/Thr^382/383^ by western blot. KIF7-CC over-expression increased both nuclear and cytoplasmic LKB1 expression in PC3, C4-2B and 22Rv1 cells by western blot. As PTEN was slightly expressed in LNCaP-derived C4-2B cells, increasing protein amount and extending exposure are needed for PTEN detection in C4-2B cells. **(B)** correlation of *KIF7* with *LKB1* from cBioPortal. Downregulation of LKB1 by shRNAs restored the inhibitory effect of KIF7-CC on AKT phosphorylation at Ser^473^ **(C)** cell proliferation **(D)** and colony formation **(E)** in PC3, C4-2B and 22Rv1 cells by western blot, cell number counting as well as colony formation assays, respectively. *, *p* < 0.05; **, *p* < 0.01; ***, *p* < 0.001, compared with KIF7-CC shNTC, mean ± SD was presented.

The AKT pathway is important for cell proliferation, migration and survival, which is aberrantly activated in PCa [22] as well as in resistance to taxane [23]. Liver kinase B1 (LKB1) inhibits AKT activity through PTEN activation [24, 25]. We then investigated the effects of KIF7-CC in LKB1/PTEN/AKT signaling cascade. Western blot revealed that phosphorylation of AKT at Ser^473^ was significantly attenuated when KIF7-CC was overexpressed in PC3, C4-2B and 22Rv1 cells. And KIF7-CC increased LKB1 and PTEN expression as well as PTEN activity by increasing phosphorylation of PTEN at Ser^380^/Thr^382/383^ in these cells, except in PC3, which is a PTEN-negative cell line [26] (Figure 5A).

We next investigated the mechanisms of regulation of LKB1 signaling by KIF7-CC. LKB1 signaling is regulated through 3 main mechanisms: 1) phosphorylation, 2) subcellular localization, and 3) formation of complexes with STE20-related adaptor-α (STRADα) and calcium binding protein 39 (CAB39/MO25α). STRADα determines the subcellular localization of LKB1 by initiating its translocation from the nucleus to the cytoplasm [27]. CAB39/MO25α stabilizes the LKB1-STRADα complex, thus further increasing the catalytic activity of LKB1 [28]. KIF7-CC-overexpressing cells significantly enhanced phosphorylation of LKB1 at Ser^428^ (Figure 5A). To check the subcellular localization of LKB1, we isolated the nuclear and cytosolic fractions from the KIF7-CC and control cells. LKB1 expression was increased in both nucleus and cytosol of the KIF7-CC cells compared with those of the controls from PC3, C4-2B and 22Rv1 cells (Figure 5A). In addition, we also checked STRADα and MO25α expression in the KIF7-CC-overexpressing cells compared with that in controls by western blot, and we found no difference in their expression (data not shown). These data imply that KIF7-CC increases both nuclear and cytosolic LKB1 expression as well as induces LKB1 phosphorylation at Ser^428^, which activates LKB1 signaling.

According to the different cohorts from cBioPortal, *KIF7* expression was found to be lowly and moderately correlated with *LKB1* expression in the Cancer Genome Atlas Research Network [29] (Pearson correlation coefficient: 0.38, n = 333) and Baca *et al*. [30] (Pearson correlation coefficient: 0.61, n = 16) cohorts, respectively (Figure 5B). To explore whether LKB1 mediates the inhibitory effects of KIF7-CC in PCa, KIF7-CC-overexpressing PC3, C4-2B and 22Rv1 cells were lentivirally transduced with either LKB1 short hairpin RNAs (shRNAs: shH and shM) or a non-target control (shNTC) sequence to generate stably repressed cells or control cells. Stable LKB1 knockdown was confirmed by western blot (Figure 5C). Phosphorylation of AKT at Ser^473^ was restored to the control levels when LKB1 was knocked down in the KIF7-CC-overexpressing PC3, C4-2B and 22Rv1 cells (Figure 5C), which suggested that the inhibitory effect of AKT phosphorylation by KIF7-CC might through LKB1. Knockdown of LKB1 also restored proliferation (Figure 5D) and colony formation (Figure 5E).

**Figure 6:**
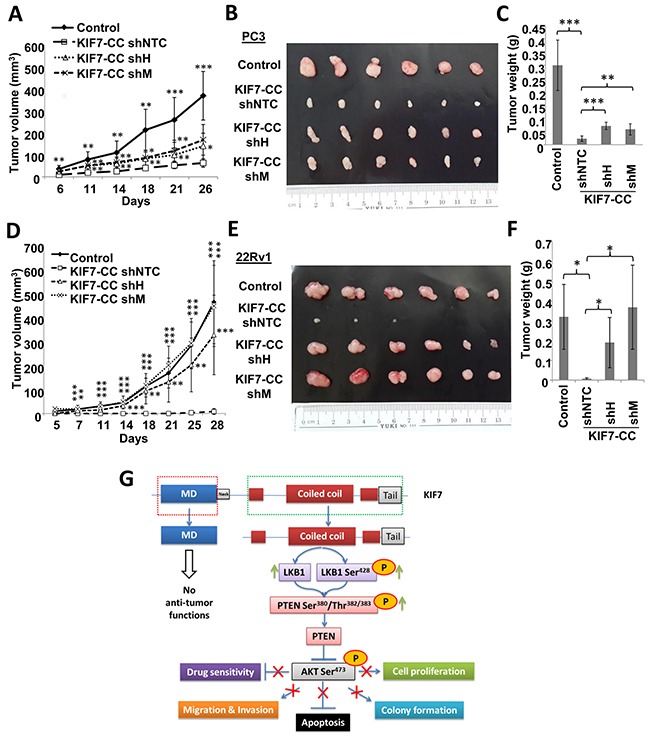
LKB1 mediated the inhibitory effects of KIF7-CC on the growth of PCa cell-derived tumor in nude mice Stable knockdown of LKB1 by shRNAs and the non-target control (shNTC) in PC3 and 22Rv1 KIF7-CC over-expression cells together with their parental controls were subcutaneously implanted into the nude mice. **(A&D)** tumor growth was monitored at various time points. **(B&E)** representative tumor images from PC3 and 22Rv1 cell-derived xenografts were shown. **(C&F)** bar-char summarizing the tumor weights from different groups; *, *p* < 0.05; **, *p* < 0.01; ***, *p* < 0.001, compared with the KIF7-CC shNTC group. Data represent the mean ± SD of different group, there are 6 mice in each group. **(G)** schematic representation depicting inhibitory effect of KIF7-CC on AKT through LKB1/PTEN signaling.

To further support our *in vitro* findings, we next evaluated the effect of LKB1 on tumorigenesis in KIF7-CC-overexpressing xenografts. Mice injected with LKB1-repressed clones formed more tumors, and the tumors were larger and heavier than NTC cells when KIF7-CC was over-expressed in PC3 or 22Rv1 cells (Figure 6A–6F). Notably, in the 22Rv1 xenografts, knockdown of LKB1 attenuated the inhibitory effect of KIF7-CC on tumor growth and restored tumor volumes similar to those found in control xenografts.

Taken together, these results suggest that KIF7-CC might elicit its growth-inhibitory effects through inhibition of AKT activation, which is partially through increasing LKB1/PTEN in PCa cells (Figure 6G).

## DISCUSSION

Widespread expression of *KIF7* is found in normal tissues (Supplementary Figure 5). To the best of our knowledge, this is the first report on the expression and functional role of KIF7 in human solid tumors. Until recently, only two studies have been reported to assess the role of KIF7 in carcinogenesis. Li *et al*. found that Kif7 cooperative with Sufu inhibited basal cell carcinoma in a transgenic mouse model [31]. Ho *et al*. elucidated that KIF7 attenuated proliferation and migration in choriocarcinoma cells *in vitro* [32]. However, no direct evidence was provided indicating the role of KIF7 in human cancer development. We showed for the first time that KIF7-CC but not KIF7-MD acted as a tumor suppressor in PCa. Kinesins are divided into mitotic and non-mitotic kinesins [33]. Mitotic kinesins are crucial for spindle formation during mitosis, which are targets for chemotherapeutic intervention [34]. KIF7 is one of the non-mitotic kinesins involved in cilia function and it is a mammalian homologue of the *Drosophila melanogaster* kinesin-like protein Costal2, which is a key regulator in Hh signaling [35]. To date, the effects of KIF7 on the Hh pathway are still controversial. Several groups have shown that Kif7 inhibits Hh signaling in mouse embryos, embryonic fibroblasts [12] and lung fibroblasts [36]. Moreover, Kif7 acts downstream of Smo and upstream of Gli2 and has both negative and positive roles in Hh signal transduction [37]. However, Varjosalo *et al* demonstrated that unlike Costal2 from *Drosophila* Kif7 has no effect on Hh pathway in NIH-3T3 cells [38]. Our data illustrate that no difference has been detected in the mRNA levels of Hh signaling targets (*Gli1, PTCH1, NKX2.2, IGF2* and *CyclinD2*) in PC3, C4-2B and 22Rv1 cells overexpressing KIF7-CC compared with controls according to qPCR (data not shown). This indicates that KIF7-CC does not affect Hh signaling in PCa *in vitro*.

PI3K/AKT signaling is prominently involved in PCa progression, and PTEN is mutated in 30-60% of PCa [39]. Several groups have shown that LNCaP and DU145 but not PC3 cells express *PTEN* mRNA according to northern blot [26, 40] and RT-PCR [26, 41]. PTEN is highly expressed in DU145 and 22Rv1 cells and slightly expressed in LNCaP as well as its derivative C4-2B cells, while no PTEN is found in PC3 cells according to our western blot (Supplementary Figure 6). Phosphorylation of AKT at Ser^473^ has been identified as a biomarker for PCa and has been correlated with a higher probability of disease recurrence [42]. Importantly, LKB1 is thought to function as a tumor suppressor to inhibit AKT pathway through PTEN stabilization and activation [43–45]. We identified that LKB1 was involved in the inhibitory effect of KIF7 on AKT phosphorylation at Ser^473^. It was interesting that Lkb1 also localized in the cilia in mouse embryonic fibroblasts [46] and controlled the length of primary cilia [47], which had the same effect as Kif7 [12], raising the possibility that LKB1 might play an important role in the tumor suppressive effect of KIF7. According to two different PCa cohorts from cBioPortal, *KIF7* and *LKB1* are found to be co-expressed (Figure 5B). Furthermore, we identified that KIF7-CC increased LKB1 expression and its phosphorylation at Ser^428^ in PCa cells, which induced LKB1 to bind and phosphorylate PTEN at Ser^380^/Thr^382/383^ and then inhibited AKT phosphorylation at Ser^473^ (Figure 5). The anti-proliferation and anti-tumor functions of KIF7-CC through LKB1 were further validated when LKB1 was downregulated in KIF7-CC-overexpressing PCa cells, resulting in the abolition of these effects (Figure 5 &6). To our knowledge, this is the first report to provide well-validated data showing that the LKB1/PTEN/AKT pathway mediates the tumor suppressive effect of KIF7-CC in PCa. Despite this information, the detailed mechanisms underlying KIF7-CC-mediated LKB1 expression and phosphorylation at Ser^428^ need to be further investigated. Down-regulation of *KIF7* is not only restricted to PCa but also present in other cancer types (Supplementary Figure 3). Whether KIF7 plays anti-cancer effect in other cancers needs more convincing evidence.

Although novel hormonal agents and immunology-based therapies have shown improvements in survival, taxane-based therapy will continue to be a viable option for patients with metastatic castrate-resistant disease for the foreseeable future. KIF7-CC overexpression significantly sensitized the PCa cells to paclitaxel, which led to new strategies to enhance the efficacy of taxane-based treatments for patients with advanced disease. The results from on-going and future trials will guide the development of KIF7 overexpression by adenovirus and will be important for assessing its full potential as a single agent or in combination therapy with taxane-based treatments.

In summary, our present study showed that KIF7-CC but not KIF7-MD could modulate the LKB1/PTEN/AKT pathway, thereby suppressing PCa cell proliferation, colony formation, migration and invasion as well as sensitizing the cells to paclitaxel treatment *in vitro* and inhibiting prostate carcinogenesis *in vivo*. Our results revealed that under-expression of KIF7 in PCa patients might contribute to paclitaxel resistance through reduction of LKB1 or PTEN expression and enhanced activation of AKT. Taken together, this evidence suggests that KIF7 might represent a novel therapeutic target in PCa.

## MATERIALS AND METHODS

### Clinical samples

109 FFPE prostate specimens were selected from the Department of Pathology, Queen Mary Hospital, The University of Hong Kong. They comprised of 8 normal, 17 BPH, 13 PIN, and 71 PCa specimens. Tissue samples from patients who had undergone hormone deprivation or radiation therapy were excluded. Archival dates of the specimens fell between 1997 and 2004. The study was approved by the Ethics Committee of the University of Hong Kong. Histological diagnoses were made and tumors were graded by a pathologist (KW Chan) according to the GS method. Clinical information of the patients is summarized in Supplementary Table 1.

### Immunohistochemistry (IHC) and evaluation of IHC staining

All 109 FFPE prostate tissues were used to construct a TMA. Each specimen was sampled in triplicate to account for tumor heterogeneity (n = 327). IHC was performed and the stained sections were reviewed by KW Chan as described [48].

### Cell culture and drug treatments

PCa cell lines LNCaP, PC3 and DU145 were obtained from the American Type Culture Collection (Rockville, MD). 22Rv1 and C4-2B were gifts from Professor Franky Chan of the Chinese University of Hong Kong. Immortalized, normal human prostate epithelial cell HPr-1 was established in our laboratory [19]. The immortalized, normal human prostate epithelial cell NPTX was a gift by Drs. Robert Bright and Susan Topalian of NCI, NIH, Bethesda, MD [49]. All appropriate human cell lines were authenticated by the Centre for Genomic Sciences, The University of Hong Kong, using an AmpFISTR Identifier^®^ PCR Amplification kit (Applied Biosystems, Foster City, CA) (Supplementary Table 2). For drug treatments, 5-AZA-dC was added at a concentration of 5 μM every day. TSA treatment was performed at a concentration of 300 nM for one day. Cells were treated with paclitaxel from 0 to 30 nM for 3 days. All drugs were purchased from Sigma.

### RNA isolation, cDNA synthesis and Quantitative real-time PCR

FFPE PCa and normal tissue blocks were microdissected for RNA extraction using a Pinpoint™ Slide RNA Isolation System II kit (Zymo Research, Orange, CA). RNA isolation from cells, cDNA synthesis and quantitative real-time polymerase chain reaction (qPCR) were performed as described [48]. Primers are listed in Supplementary Table 3.

### Bisulfite genomic sequencing (BGS)

Genomic DNA was extracted from cell lines and frozen tissues using DNAzol^®^ (Invitrogen, Carlsbad, CA). BGS was performed as described [50]. The primers are listed in Supplementary Table 3.

### Western blot

Antibody details are listed in the Supplementary Table 4.

### Cell proliferation and colony formation

For proliferation measurement, 20,000 cells per well were seeded into 24-well plate. For colony formation, 500 cells per well were seeded into 6-well plate and cultured for 2 weeks. At the end of the experiment, media were removed and cells were fixed with methanol followed by staining with 0.01% crystal violet.

### Cell migration and invasion

Cell migration (2×10^4^ cells, 16 hours) or invasion (2×10^5^ cells, 36 hours) assay was performed using 8 μM-pore size transwell^®^ insert (Costar) coated without or with matrigel (BD Pharmingen), respectively. Cells were seeded in the upper chamber of the transwell with serum-free medium. The lower chamber contained normal culture medium. Migrated or invaded cells were fixed with methanol and stained with crystal violet. Three independent views of the transwell membrane were photographed and number of cells was counted.

### Drug sensitivity assay

Drug sensitivity assay was performed by MTT test and apoptosis was quantified by Annexin V-FITC and PI staining kit from BD Pharmingen^TM^ (San Jose, CA).

### Generation of PCa cells with stable over-expression of KIF7-CC and KIF7-MD

The expression plasmid pEF6/V5-His-KIF7-CC was generated by subcloning KIF7-CC from pCR-BluntII-TOPO-KIF7 (Imagenes) into pEF6/V5-His-TOPO (Invitrogen, Carlsbad, CA). PC3, C4-2B and 22Rv1 cells were transfected with pEF6/V5-His-KIF7-CC or the control vector using lipofectamine 2000 (Invitrogen, Carlsbad, CA). Stable clones were selected by blasticidin S (10 μg/ml, sigma).

The KIF7-MD was cloned into a pCDH-CMV-MCS-EF1-copGFP vector (System Biosciences, Mountain View, CA). PC3 and C4-2B cells were transduced with lentivirus packaged with either a KIF7-MD-expressing vector or an empty vector to generate KIF7-MD-overexpressing (MD) or control cells (GFP) using a Lenti Starter kit (SBI, Mountain View, CA). GFP-positive cells were selected by fluorescence-activated cell sorting using BD Aria (BD Biosciences).

### Stable knockdown of LKB1 by lentivirus-mediated short hairpin RNA in KIF7-CC over-expression cells

LKB1 knockdown in the KIF7-CC over-expression clones from PC3, C4-2B and 22Rv1 cells was performed by infection with lentivirus that expressed human LKB1-specific short hairpin RNAs (shH and shM) (61231 & 61242) or non-target control shRNA (shNTC) (1864), which were packaged with psPAX2 (12260) and pMD2.G (12259) from Addgene (Cambrige, MA; http://www.addgene.org) using lipofectamine 2000. Infected cells were selected by puromycin (2 μg/ml, Sigma).

### *In vivo* tumorigenicity in nude mice

All animal experiments were approved and performed according to guidelines by the Animal Ethics Committee, The University of Hong Kong. Briefly, 2×10^5^ of PC3 or 22Rv1 cells or 10^6^ of C4-2B cells were injected subcutaneously into the 6-week male athymic BALB/c nu/nu mice and tumors were measured as described previously [48].

### Statistical methods

Differences between groups were analyzed by student's *t* test for continuous variables. Correlation between clinicopathological variables and KIF7 expression were analyzed by χ^2^ test or Fisher Exact tests. All statistical tests were analyzed by SPSS 18 for Windows (SPSS Inc., Chicago, IL). A 2-tailed *p* value of < 0.05 was considered statistically significant.

## SUPPLEMENTARY MATERIALS FIGURES AND TABLES





## References

[1] Torre LA, Bray F, Siegel RL, Ferlay J, Lortet-Tieulent J, Jemal A (2015). Global cancer statistics, 2012. CA Cancer J Clin.

[2] Siegel RL, Miller KD, Jemal A (2015). Cancer statistics, 2015. CA Cancer J Clin.

[3] Sharifi N, Dahut WL, Steinberg SM, Figg WD, Tarassoff C, Arlen P, Gulley JL (2005). A retrospective study of the time to clinical endpoints for advanced prostate cancer. BJU Int.

[4] Beer TM, El-Geneidi M, Eilers KM (2003). Docetaxel (taxotere) in the treatment of prostate cancer. Expert Rev Anticancer Ther.

[5] Holohan C, Van Schaeybroeck S, Longley DB, Johnston PG (2013). Cancer drug resistance: an evolving paradigm. Nat Rev Cancer.

[6] Freedland SJ, Aronson WJ (2004). Examining the relationship between obesity and prostate cancer. Rev Urol.

[7] Baillargeon J, Rose DP (2006). Obesity, adipokines, and prostate cancer (review). Int J Oncol.

[8] Mok CA, Heon E, Zhen M (2010). Ciliary dysfunction and obesity. Clin Genet.

[9] Waters AM, Beales PL (2011). Ciliopathies: an expanding disease spectrum. Pediatr Nephrol.

[10] Mans DA, Voest EE, Giles RH (2008). All along the watchtower: is the cilium a tumor suppressor organelle?. Biochim Biophys Acta.

[11] Hassounah NB, Nagle R, Saboda K, Roe DJ, Dalkin BL, McDermott KM (2013). Primary cilia are lost in preinvasive and invasive prostate cancer. PLoS One.

[12] He M, Subramanian R, Bangs F, Omelchenko T, Liem KF, Kapoor TM, Anderson KV (2014). The kinesin-4 protein Kif7 regulates mammalian Hedgehog signalling by organizing the cilium tip compartment. Nat Cell Biol.

[13] Cheung HO, Zhang X, Ribeiro A, Mo R, Makino S, Puviindran V, Law KK, Briscoe J, Hui CC (2009). The kinesin protein Kif7 is a critical regulator of Gli transcription factors in mammalian hedgehog signaling. Sci Signal.

[14] Staub E, Grone J, Mennerich D, Ropcke S, Klamann I, Hinzmann B, Castanos-Velez E, Mann B, Pilarsky C, Brummendorf T, Weber B, Buhr HJ, Rosenthal A (2006). A genome-wide map of aberrantly expressed chromosomal islands in colorectal cancer. Mol Cancer.

[15] Signoretti S, Waltregny D, Dilks J, Isaac B, Lin D, Garraway L, Yang A, Montironi R, McKeon F, Loda M p63 is a prostate basal cell marker and is required for prostate development. Am J Pathol.

[16] Parsons JK, Gage WR, Nelson WG, De Marzo AM p63 protein expression is rare in prostate adenocarcinoma: implications for cancer diagnosis and carcinogenesis. Urology.

[17] Gulzar ZG, McKenney JK, Brooks JD (2013). Increased expression of NuSAP in recurrent prostate cancer is mediated by E2F1. Oncogene.

[18] Esteller M (2008). Epigenetics in cancer. N Engl J Med.

[19] Choo CK, Ling MT, Chan KW, Tsao SW, Zheng Z, Zhang D, Chan LC, Wong YC (1999). Immortalization of human prostate epithelial cells by HPV 16 E6/E7 open reading frames. Prostate.

[20] Klejnot M, Kozielski F (2012). Structural insights into human Kif7, a kinesin involved in Hedgehog signalling. Acta Crystallogr D Biol Crystallogr.

[21] Hudes GR, Nathan F, Khater C, Haas N, Cornfield M, Giantonio B, Greenberg R, Gomella L, Litwin S, Ross E, Roethke S, McAleer C (1997). Phase II trial of 96-hour paclitaxel plus oral estramustine phosphate in metastatic hormone-refractory prostate cancer. J Clin Oncol.

[22] Li B, Sun A, Jiang W, Thrasher JB, Terranova P (2014). PI-3 kinase p110beta: a therapeutic target in advanced prostate cancers. Am J Clin Exp Urol.

[23] Liu Z, Zhu G, Getzenberg RH, Veltri RW (2015). The Upregulation of PI3K/Akt and MAP Kinase Pathways is Associated with Resistance of Microtubule-Targeting Drugs in Prostate Cancer. J Cell Biochem.

[24] Song P, Wu Y, Xu J, Xie Z, Dong Y, Zhang M, Zou MH (2007). Reactive nitrogen species induced by hyperglycemia suppresses Akt signaling and triggers apoptosis by upregulating phosphatase PTEN (phosphatase and tensin homologue deleted on chromosome 10) in an LKB1-dependent manner. Circulation.

[25] Vazquez F, Ramaswamy S, Nakamura N, Sellers WR (2000). Phosphorylation of the PTEN tail regulates protein stability and function. Mol Cell Biol.

[26] Whang YE, Wu X, Suzuki H, Reiter RE, Tran C, Vessella RL, Said JW, Isaacs WB, Sawyers CL (1998). Inactivation of the tumor suppressor PTEN/MMAC1 in advanced human prostate cancer through loss of expression. Proc Natl Acad Sci U S A.

[27] Baas AF, Boudeau J, Sapkota GP, Smit L, Medema R, Morrice NA, Alessi DR, Clevers HC (2003). Activation of the tumour suppressor kinase LKB1 by the STE20-like pseudokinase STRAD. EMBO J.

[28] Boudeau J, Baas AF, Deak M, Morrice NA, Kieloch A, Schutkowski M, Prescott AR, Clevers HC, Alessi DR (2003). MO25alpha/beta interact with STRADalpha/beta enhancing their ability to bind, activate and localize LKB1 in the cytoplasm. EMBO J.

[29] Cancer Genome Atlas Research Network (2015). The Molecular Taxonomy of Primary Prostate Cancer. Cell.

[30] Baca SC, Prandi D, Lawrence MS, Mosquera JM, Romanel A, Drier Y, Park K, Kitabayashi N, MacDonald TY, Ghandi M, Van Allen E, Kryukov GV, Sboner A (2013). Punctuated evolution of prostate cancer genomes. Cell.

[31] Li ZJ, Nieuwenhuis E, Nien W, Zhang X, Zhang J, Puviindran V, Wainwright BJ, Kim PC, Hui CC (2012). Kif7 regulates Gli2 through Sufu-dependent and -independent functions during skin development and tumorigenesis. Development.

[32] Ho J, Du Y, Wong OG, Siu MK, Chan KK, Cheung AN (2014). Downregulation of the gli transcription factors regulator Kif7 facilitates cell survival and migration of choriocarcinoma cells. PLoS One.

[33] Rath O, Kozielski F (2012). Kinesins and cancer. Nat Rev Cancer.

[34] Huszar D, Theoclitou ME, Skolnik J, Herbst R (2009). Kinesin motor proteins as targets for cancer therapy. Cancer Metastasis Rev.

[35] Forbes AJ, Nakano Y, Taylor AM, Ingham PW (1993). Genetic analysis of hedgehog signalling in the Drosophila embryo. Dev Suppl.

[36] Coles GL, Baglia LA, Ackerman KG (2015). KIF7 Controls the Proliferation of Cells of the Respiratory Airway through Distinct Microtubule Dependent Mechanisms. PLoS Genet.

[37] Liem KF, He M, Ocbina PJ, Anderson KV (2009). Mouse Kif7/Costal2 is a cilia-associated protein that regulates Sonic hedgehog signaling. Proc Natl Acad Sci U S A.

[38] Varjosalo M, Li SP, Taipale J (2006). Divergence of hedgehog signal transduction mechanism between Drosophila and mammals. Dev Cell.

[39] Li J, Yen C, Liaw D, Podsypanina K, Bose S, Wang SI, Puc J, Miliaresis C, Rodgers L, McCombie R, Bigner SH, Giovanella BC, Ittmann M (1997). PTEN, a putative protein tyrosine phosphatase gene mutated in human brain, breast, and prostate cancer. Science.

[40] Bastola DR, Pahwa GS, Lin MF, Cheng PW (2002). Downregulation of PTEN/MMAC/TEP1 expression in human prostate cancer cell line DU145 by growth stimuli. Mol Cell Biochem.

[41] Vlietstra RJ, van Alewijk DC, Hermans KG, van Steenbrugge GJ, Trapman J (1998). Frequent inactivation of PTEN in prostate cancer cell lines and xenografts. Cancer Res.

[42] Kreisberg JI, Malik SN, Prihoda TJ, Bedolla RG, Troyer DA, Kreisberg S, Ghosh PM (2004). Phosphorylation of Akt (Ser473) is an excellent predictor of poor clinical outcome in prostate cancer. Cancer Res.

[43] Jimenez AI, Fernandez P, Dominguez O, Dopazo A, Sanchez-Cespedes M (2003). Growth and molecular profile of lung cancer cells expressing ectopic LKB1: down-regulation of the phosphatidylinositol 3’-phosphate kinase/PTEN pathway. Cancer Res.

[44] Pearson HB, McCarthy A, Collins CM, Ashworth A, Clarke AR (2008). Lkb1 deficiency causes prostate neoplasia in the mouse. Cancer Res.

[45] Yoshimoto M, Cunha IW, Coudry RA, Fonseca FP, Torres CH, Soares FA, Squire JA (2007). FISH analysis of 107 prostate cancers shows that PTEN genomic deletion is associated with poor clinical outcome. Br J Cancer.

[46] Boehlke C, Kotsis F, Patel V, Braeg S, Voelker H, Bredt S, Beyer T, Janusch H, Hamann C, Godel M, Muller K, Herbst M, Hornung M (2010). Primary cilia regulate mTORC1 activity and cell size through Lkb1. Nat Cell Biol.

[47] Jacob LS, Wu X, Dodge ME, Fan CW, Kulak O, Chen B, Tang W, Wang B, Amatruda JF, Lum L (2011). Genome-wide RNAi screen reveals disease-associated genes that are common to Hedgehog and Wnt signaling. Sci Signal.

[48] Ma S, Chan YP, Kwan PS, Lee TK, Yan M, Tang KH, Ling MT, Vielkind JR, Guan XY, Chan KW (2011). MicroRNA-616 induces androgen-independent growth of prostate cancer cells by suppressing expression of tissue factor pathway inhibitor TFPI-2. Cancer Res.

[49] Bright RK, Vocke CD, Emmert-Buck MR, Duray PH, Solomon D, Fetsch P, Rhim JS, Linehan WM, Topalian SL (1997). Generation and genetic characterization of immortal human prostate epithelial cell lines derived from primary cancer specimens. Cancer Res.

[50] Liu J, Lam JB, Chow KH, Xu A, Lam KS, Moon RT, Wang Y (2008). Adiponectin stimulates Wnt inhibitory factor-1 expression through epigenetic regulations involving the transcription factor specificity protein 1. Carcinogenesis.

