# Supplementary material for: KIF7 attenuates prostate tumor growth through LKB1-mediated AKT inhibition
Source: Oncotarget. 2017 Apr 26;8(33):54558–71. doi: 10.18632/oncotarget.17421 (PMC5589603; doi:10.18632/oncotarget.17421)
Supplement: Supplementary file 1 [file oncotarget-08-54558-s001.pdf]

KIF7 attenuates prostate tumor growth through LKB1-mediated AKT inhibition

SUPPLEMENTARY MATERIALS AND METHODS

Normal tissues cDNA

Human normal tissue cDNA was purchased from Clontech (Mountain View, CA, USA).

SUPPLEMENTARY FIGURES AND TABLES

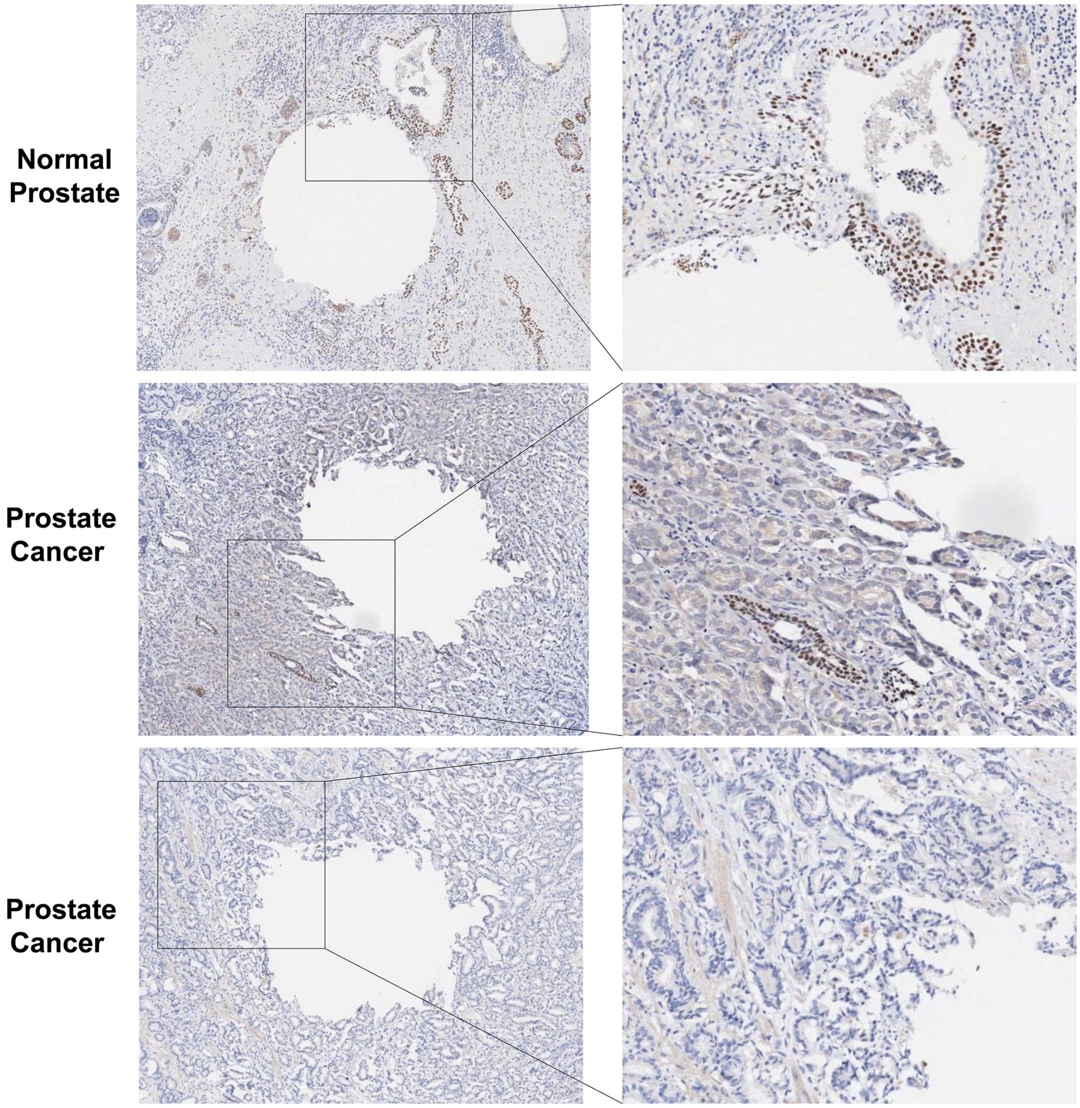

Supplementary Figure 1: p63 expression in normal prostates and prostate cancer tissues by immunohistochemistry. p63 was highly expressed in normal prostate epithelium, while rarely expressed in prostate adenocarcinoma cells. Holes in the tissues are the microdissection sites with 1.0 mm punch.

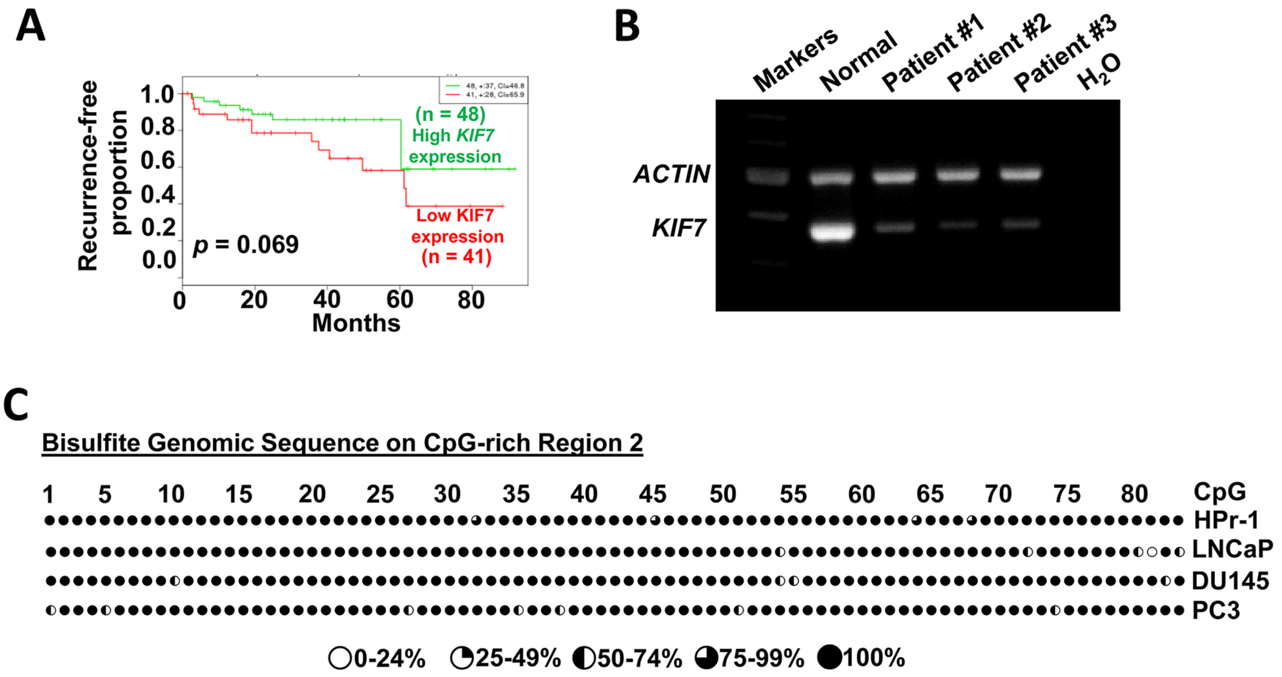

Supplementary Figure 2: Expression of *KIF7* and its promoter methylation in CpG-rich region 2. (A) Kaplan-Meier recurrence analysis of *KIF7* expression in Gulzar Z *et al.* dataset, $p = 0.069$, log rank test; **(B)** multiplex RT-PCR of *KIF7* in one normal prostate and three PCa tissues, with *β-ACTIN* as an internal control; **(C)** bisulfite genomic sequence on CpG-rich region 2 of *KIF7* promoter in HPr-1, LNCaP, DU145 and PC3 cells.

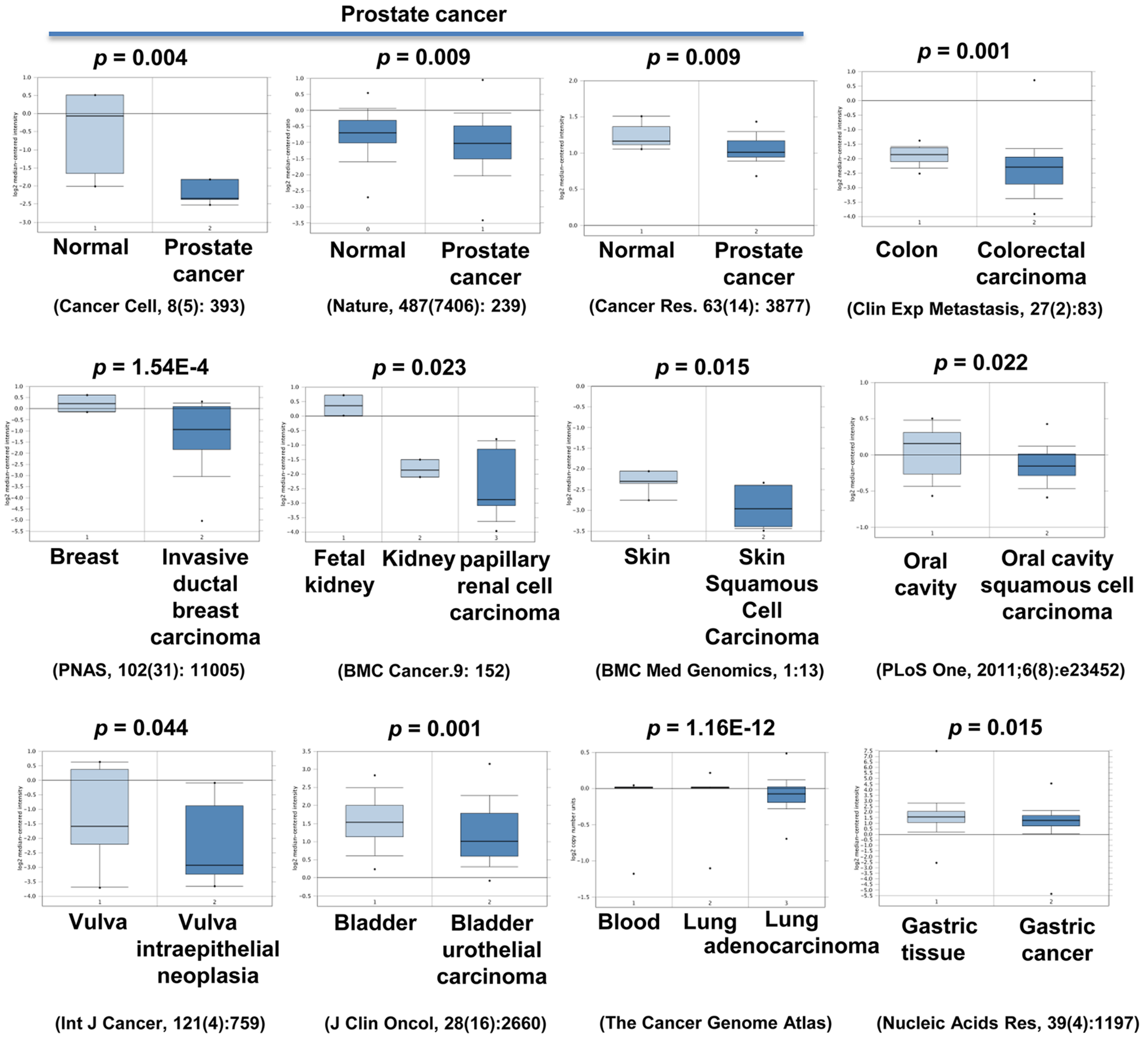

Supplementary Figure 3: Expression of *KIF7* from Oncomine. *KIF7* was significantly down-regulated in human prostate cancer and other cancers by Oncomine. Box and whisker plots of Oncomine data on *KIF7* mRNA levels (expressed as the log2 median-centered intensity) in various normal and cancerous tissues. *p* values, Student's *t* test.

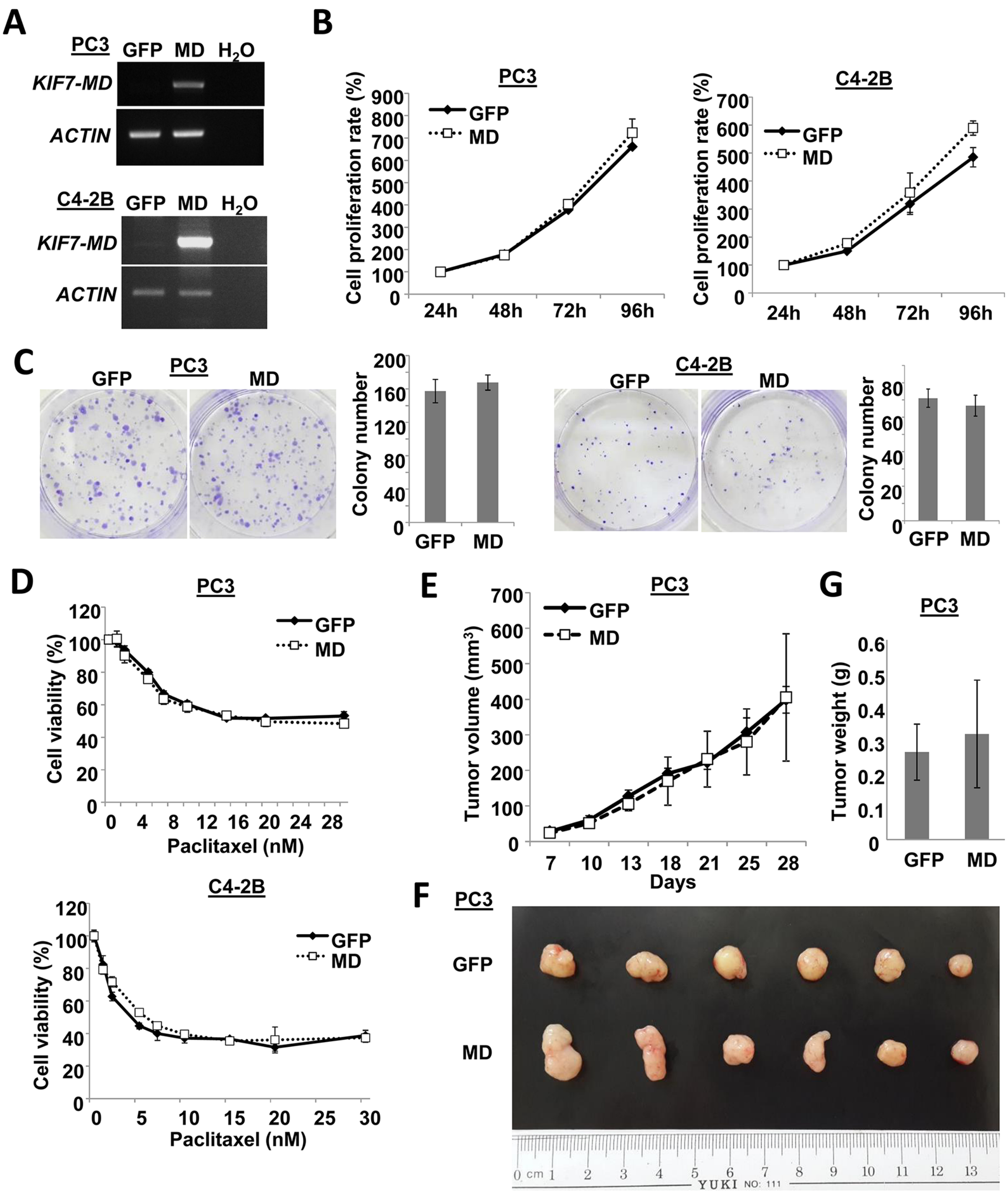

Supplementary Figure 4: No anti-tumor functions had been found in the motor domain of KIF7 in prostate carcinogenesis. (A) motor domain (MD) of KIF7 was stably over-expressed in PC3 and C4-2B cells through lentivirus–based approach as described in the Materials and Methods. Multiplex RT-PCR analysis of the *KIF7-MD* over-expression in these cells compared with GFP lentivirus controls. *β-ACTIN* was amplified as an internal control. KIF7-MD had no effects on cell proliferation **(B)** colony formation **(C)** as well as paclitaxel sensitivity **(D)** in PC3 and C4-2B cells compared with GFP controls. No inhibitory effects of KIF7-MD on the growth of PC3 prostate cancer cell-derived tumor in nude mice. **(E)** tumor growth was monitored by measuring the visible tumor sizes at various time points. **(F)** representative tumor images from PC3 cell-derived xenografts were shown. **(G)** bar-char summarizing the tumor weights from different groups, no significance had been found between the groups. Data represent the mean ± SD of different group, there are 6 mice in each group.

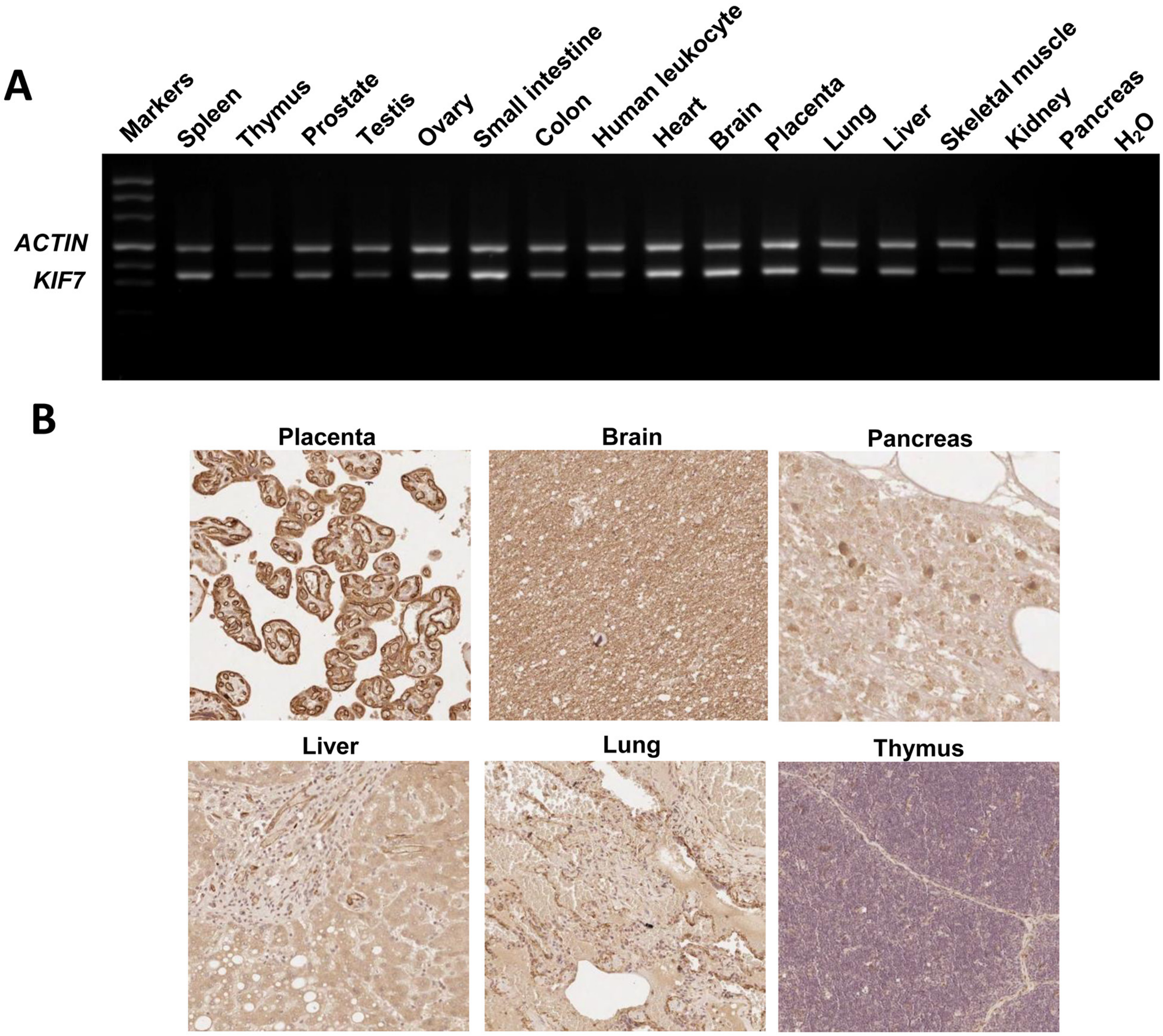

Supplementary Figure 5: Expression of KIF7 in normal tissues. (A) multiplex semi-quantitative PCR analysis of *KIF7* in normal tissues, with *β-ACTIN* as an internal control. **(B)** KIF7 protein expression in normal tissues by IHC.

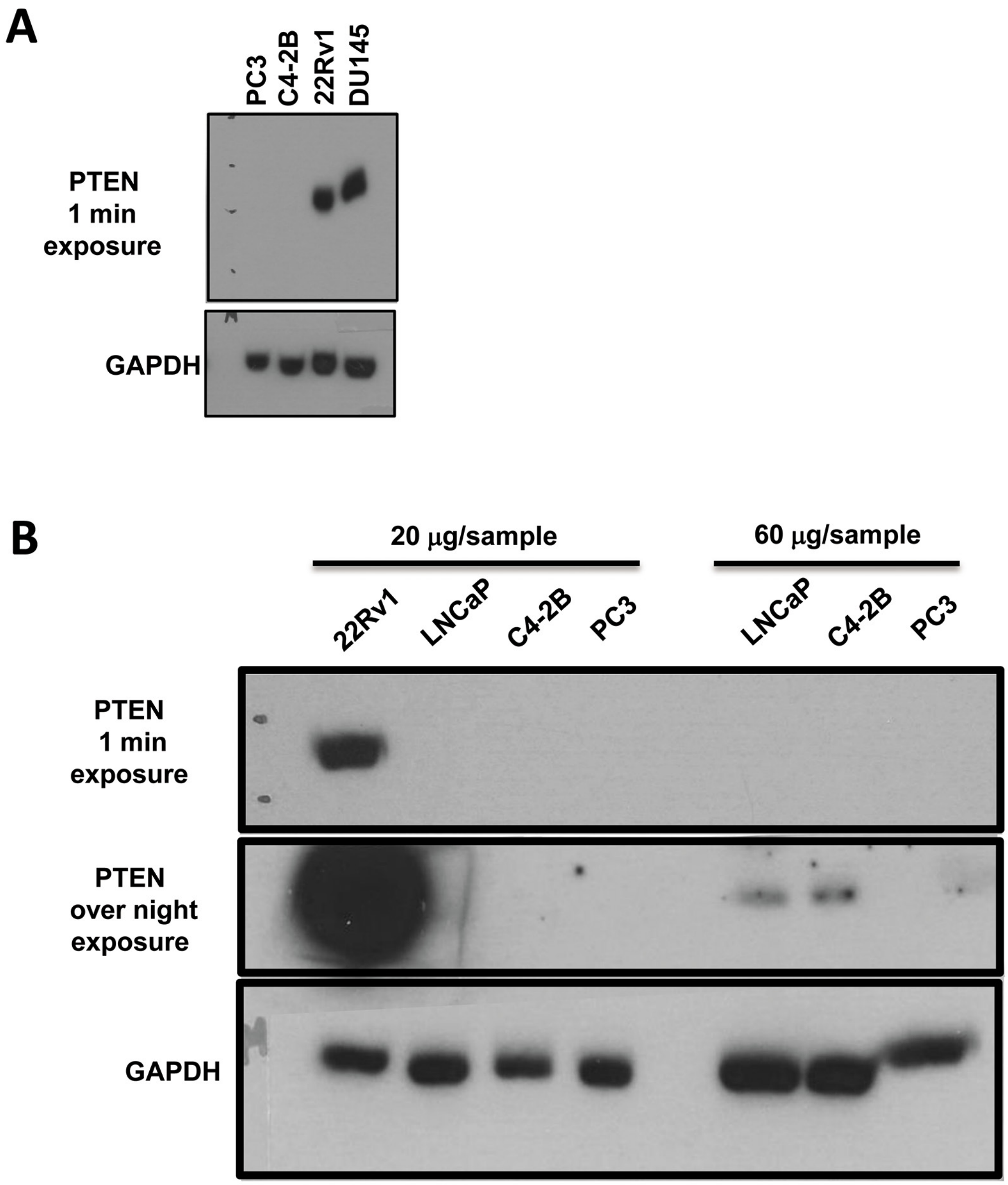

Supplementary Figure 6: Expression of PTEN in prostate cancer cell lines. **(A)** Expression of PTEN in PC3, C4-2B, 22Rv1 and DU145 cells by western blot (20 µg/sample). **(B)** Expression of PTEN was detected in 22Rv1 (20 µg), LNCaP (20 µg), C4-2B(20 µg), PC3 (20 µg), LNCaP (60 µg), C4-2B (60 µg) and PC3 (60 µg) cells by western blot, with GAPDH as an internal control.

Supplementary Table 1: Clinical and pathological features of the patients

	No. of case	%	Median (range)
**Histological group**	109		
Normal	8	7	
Prostate hyperplasia	17	16	
High-grade PIN	13	12	
Prostate cancer (combined GS < 7)	26	24	
Prostate cancer (combined GS ≥ 7)	45	41	
**Age, years**			76 (35 - 94)
**Pre-PSA level, ng/mL**	54		52.5 (0.4 - 2392)
Low (PSA < 10 ng/mL)	7	13	
High (PSA ≥ 10 ng/mL)	47	87	
**Metastasis (within 24 months)**	71		
Yes	26	37	
No	45	63	
**Site of metastasis detected**	30		
Bone	27	90	
Lymph node	3	10	

Pre-PSA = pre-operative prostate-specific antigen.

Supplementary Table 2: DNA profiling of LNCaP, C4-2B, DU145, PC3 and 22Rv1

Loci	ATCC (CRL-1740) LNCaP-FGC	Submitted sample: LNCaP	Submitted sample: C4-2B*	ATCC (HTB-81) DU145	Submitted sample: DU145	ATCC (CRL-1435) PC3	Submitted sample: PC3	ATCC (CRL-2505) 22Rv1	Submitted sample: 22Rv1
AMEL	X, Y	X, Y	X	X, Y	X, Y	X	X	X, Y	X, Y
CSF1PO	10, 11	10, 11	10, 11, 12	10, 11	9, 10, 11	11	11	10, 11	10, 11
D13S317	10,12	9, 10, 13	10, 11	12,13,14	12,13,14	11	11	9, 12	9, 12
D16S539	11	11, 12	10, 11	11, 13	11, 12, 13	11	11	12	12
D5S818	11, 12	11, 12	10, 11, 12	10, 13	9, 10, 11, 12	13	13	11, 12, 13	11, 12, 13
D7S820	9.1, 10.3	9	9, 11	7, 10, 11	7, 10, 11	8, 11	8, 11	9, 10, 11	9, 10, 11
TH01	9	9	8, 9	7	7	6,7	6, 7	6, 9.3	6, 9.3
TPOX	8, 9	8, 9	8, 9	11	11	8, 9	8, 9	8	8
vWA	16,18	16, 17, 18	16, 17, 18	17,18,19	17,18,19	17	17	15, 21	15, 21, 22

* C4-2B is a LNCaP derivative cell line.

Supplementary Table 3: Primer lists.

See Supplementary File 1

Supplementary Table 4: Antibody lists

Antibody	Cat. No.	Resource	Dilution
anti-KIF7	HPA043145	Sigma, St. Louis, MO	WB(1:1000); IHC(1:100)
anti-phosphorylated AKT(Ser^{473})	4060	Cell Signaling, Beverly, MA	WB(1:1000)
anti-phosphorylated PTEN (Ser^{380}/$Thr^{382/383}$)	9549	Cell Signaling, Beverly, MA	WB(1:1000);
anti-PTEN	9559	Cell Signaling, Beverly, MA	WB(1:1000);
anti-AKT	9272	Cell Signaling, Beverly, MA	WB(1:1000);
anti-phosphorylated LKB1 (Ser^{428})	3482	Cell Signaling, Beverly, MA	WB(1:1000);
anti-LKB1	ab58786	Abcam Inc., Cambridge, MA	WB(1:1000);
anti-MO25 α /CAB39	2716	Cell Signaling, Beverly, MA	WB(1:1000);
anti-STRAD	sc-34102	Santa Cruz, Dallas, Texas	WB(1:1000);
anti-Lamin B	sc-6216	Santa Cruz, Dallas, Texas	WB(1:5000);
anti-GAPDH	A00192	GenScript, Piscataway, NJ	WB(1:5000);
anti-p63	ab124762	Abcam Inc., Cambridge, MA	IHC(1:1000)
