# Supplementary material for: KIF7 attenuates prostate tumor growth through LKB1-mediated AKT inhibition
Source: Oncotarget. 2017 Apr 26;8(33):54558–71. doi: 10.18632/oncotarget.17421 (PMC5589603; doi:10.18632/oncotarget.17421)
Supplement: Supplementary file 2 [file oncotarget-08-54558-s002.docx]

**Supplementary Table S3. Primer lists**

| **Gene** | **Primers** |
| --- | --- |
| ***KIF7 CC*** | F: GGAGAAGAAGCAGGCTACGG |
|  | R: GCTGCTGTAGCACCTTCTCC |
| ***KIF7 MD*** | F: ATTCTAGAATGGGGCTGGAGGCTCAGAG |
|  | R: GCCGAATTCGATGTTCTGGGCGCGGCTG |
| ***KIF7*** **BGS F1/R1** | F: GGTTTGGATGAGGTGTTGACT |
|  | R: ACCACATAAAAACCAAAAAACT |
| ***KIF7*** **BGS F2/R2** | F: GTTTTTAGGATTTTTAAAGAT |
|  | R: CCACAACCACAACCTATCCC |
| ***β-ACTIN*** RT | F: GTTGCTATCCAGGCTGTGCT |
|  | R: AGCACTGTGTTGGCGTACAG |
| ***KIF7*** q | F: CTCTGTGGTCAGCCTGGAAC |
|  | R: GCTGCTGTAGCACCTTCTCC |
| ***β-ACTIN*** q | F: TGACCCAGATCATGTTTGAGA |
|  | R: AGTCCATCACGATGCCAGT |
| ***Gli1*** q | F: CCAGGAATTTGACTCCCAAGAG |
|  | R: CAGCATGTACTGGGCTTTGAA |
| ***PTCH1*** q | F: CCAGAAAGTATATGCACTGGCA |
|  | R: GTGCTCGTACATTTGCTTGGG |
| ***IGF2*** q | F: CGTTGAGGAGTGCTGTTTCC |
|  | R: ATTGGAAGAACTTGCCCACG |
| ***NKX2.2*** q | F: TTCTACGACAGCAGCGACAA |
|  | R: TCCTTGTCATTGTCCGGTGA |
| ***CCND2*** q | F: ACCTTCCGCAGTGCTCCTA |
|  | R: CCCAGCCAAGAAACGGTCC |
